# Cataphoretic Extirpation of Living Pulps

**Published:** 1897-04

**Authors:** Vincent M. Murr

**Affiliations:** NEW YORK.


ARTICLE VI.

### CATAPHORETIC EXTIRPATION OF LIVING PULPS.

BY VINCENT M. MURR, D. D. S., NEW YORK.

When I make the statement that the most sensitive patients will allow you to perform any operation on their teeth you may deem necessary by using cataphoresis, I speak from practical experience, as the following incidents of office practice will show.

Mr. Y. presented himself at my office for treatment, and upon examination ot his mouth, I found several badly decayed teeth. Taking the right upper first bicuspid, which was extremely sensitive. I applied the rubber and made ready the volt-selector. For the negative pole, or electrode, I used the sponge dipped in warm water containing 20 per cent. salt. The positive pole, or electrode, was a plantinum pin bent into suitable shape, and laid on a pellet of cotton saturated with a 20 per cent. aqueous solution of cocaine containing one or two drops of Calvert's carbolic acid, which has proven very satisfactory, as it preserves the solution for weeks, yea, for months, if in a brown bottle and tightly corked.

As my patient was very nervous, it required thirty-five minutes to reach ten volts. Holding it there for twenty minutes, I was able to extract the living pulp without pain to the patient.

Another patient was a young woman about twenty-two years of age, with the pulp in the right lower twelfth year molar aching, the cavity being in the anterior approximal surface. After preparing in the usual way, I was twenty minutes in reaching eight volts. After fifteen minutes I was able to extract all but a small portion of the pulp from the anterior canals. I renewed the application, and in twelve minutes was able to remove the remainder of pulp.

Those of as who have been using cataphoresis seem to get the same results, but with varying amounts of electricity. I have never been able to reach fifteen volts, my average having been five and one-half volts, but as I obtain satisfactory results, I am led to believe that some dentists are pushing the voltage too far.

In my experience cataphoresis has proven successful in every case. The time required is longer, but I charge more, and the patients seem to be willing to pay more. So, both are benefited. The patient has the work done without pain, and the dentist receives a larger fee.

---

ARTICLE VII.

